# Preferences for breast cancer survivorship programs among multiracial and ethnic women

**DOI:** 10.21203/rs.3.rs-5312826/v1

**Published:** 2024-12-09

**Authors:** Lisa Anderson, Oindrila Bhattacharyya, Akia Clark, Sharnell Smith, Michael Grimm, Elizabeth Fox, Annie Trance, Bridget A. Oppong

**Affiliations:** The Ohio State University Wexner Medical Center; The James Cancer Hospital; The Ohio State University Wexner Medical Center; The Ohio State University Wexner Medical Center; West Virginia University School of Medicine; The James Cancer Hospital; The James Cancer Hospital; The Ohio State University Wexner Medical Center

**Keywords:** breast cancer, survivorship, support services, race

## Abstract

**Purpose:**

With advancements in breast cancer treatment, survivorship has increased, leading to 3.8 million survivors in the US. These women have diverse supportive care needs, often addressed through Survivorship Programs (SPs), which provide clinical and non-clinical support services. SPs aim to deliver a holistic approach to comprehensive breast cancer treatment and recurrence prevention. Historically, disparities in SP utilization exist among minority and elderly women. This study aims to explore trends varying in SP participation by age and race within a single institution.

**Methods:**

A retrospective analysis of breast cancer patients’ survivorship needs at the James Comprehensive Cancer Center was conducted. Data were collected from JamesCare for Life programs (2019–2022), including demographics and referrals to clinical resources such as Adolescent/Young Adult care, Fertility preservation, Palliative care, Psychosocial support, and Survivorship. Participation in non-clinical areas, including Art, Education, Exercise, Mind-Body-Spirit, and Nutrition, was also evaluated. Descriptive statistics summarized patterns based on age, race, and ethnicity.

**Results:**

From 2019–2022, 2,198 patients attended SPs, with Nutrition and Exercise being the most popular. Most attendees were 60–69 years old and White. Black attendees declined from 9.9% (2019) to 5.7% (2022). Clinical resources showed the highest referral rate to survivorship clinics. Black patients saw an increase in palliative care referrals, rising from 11% to 21%.

**Conclusion:**

Data reveal differences in clinical referrals by age and race, with fewer referrals for older women and more for Black patients. Participation in non-clinical SPs was similar across groups. Future program development will focus on inclusivity and equitable access.

## Background

Although disparities persist in breast cancer outcomes, patients overall continue to benefit from advances in multimodal treatment leading to increased survival [1,2]. The 5-year survival rates for early-stage cancer are over 90%, and with rising incidence rates and aging of the U.S. population, the estimated number of cancer survivors now exceeds 3.8 million women [2,3]. As the population of survivors increases, resources addressing supportive care needs, including psychological distress and deficits in physical functioning are needed [4]. To address these deficits, many cancer centers offer a variety of programs geared towards survivors and their family members/support team [4–7].

Studies find that while interest in these survivorship programs (SPs) is generally high, the actual participation remains low. This phenomenon is a result of several barriers including lack of time (82%), work/school (65%), and lack of information about wellness activities (65%) [8]. There are disparities in engagement with support services among racial and ethnic minorities. Specifically, there are limited studies exploring the specific needs of Black breast cancer survivors, and the few that exist report a lack of culturally appropriate cancer resources necessary to help these patients understand and cope with their diagnosis [8–13]. One study found that compared to White survivors, African Americans were more likely to identify barriers related to out-of-pocket costs (28 vs. 51.6 %, p = 0.01), other health care costs (21.3 vs. 45.2 %, p = 0.01), anxiety/worry (29.4 vs. 51.6 %, p = 0.02), and transportation (4.4 vs. 16.1 %, p = 0.03) [11].

Even when survivorship programming is available, participation varies among different racial and ethnic groups. Specifically, Black survivors are less likely to utilize existing cancer support services. At our institution, 1/3 of all women referred to the free survivorship support services and programs never scheduled appointments to complete the referral [14]. To understand patients’ needs, we proposed a retrospective analysis of diverse patients’ needs and preferences in survivorship programs at the James Comprehensive Cancer Center. We evaluated the patient demographics of the participants in the James*Care* for *Life* programs, Specifically to assess patient participation by age, race and ethnicity.

## Methods

This study is a retrospective analysis of diverse breast cancer patients’ survivorship needs and preferences. The Stephanie Spielman Comprehensive Breast Center provides care for approximately 1000 analytic breast cancer cases per year. As a part of The Ohio State University Comprehensive Cancer Center – James Cancer Hospital (OSUCCC – James), a robust free survivorship support service line for breast cancer patients is provided by physicians, advanced practice providers, psychologists, dieticians, physical therapists, and social workers. Non-clinical services are also widely available to promote holistic wellness and adjustment to life with cancer. These services include cancer and survivorship education, nutrition, exercise, expressive arts, family programming, mind-body-spirit practices, disease-specific support groups, and young adult programming.

We abstracted the demographic information of participants who attended the James*Care* for *Life* programs from January 2019 through December 2022. We assessed the number of patients referred to individualized clinical resources including Adolescent/young adult care, Fertility preservation, Palliative care, Psychosocial support, and Survivorship. Survivorship is a provider-initiated visit for non-metastatic patients post treatment and includes a treatment summary, holistic needs assessment and healthy lifestyle counseling. Participation in non-clinical program areas included Art, Education, Exercise, Family, Teens, Children, Mind, Body, Spirit, Music, Nutrition, and Young Adult Survivors (ages 18–39). Descriptive statistics were utilized to summarize patterns based on age, race and ethnicity, and zip code observed within our institution. Given small samples, race categories are grouped into non-Hispanic White, Black and other.

### Data-

The Cancer Support Service Line uses an institutional quality dashboard through a data visualization tool called Tableau to monitor clinical services. Data collection of patient demographics, referral patterns, utilization trends, and encounter volumes began in July 2014 (which was the start of the academic and fiscal year) and is updated monthly through the present day. Data collected from participants who register and attend SPs were collected through Qualtrics and an internal REDCap database, funded by the National Center for Advancing Translational Sciences (Grant UL1TR001070). Data is captured by tracking registration, attendance, and post-program evaluations. Program evaluations querying demographic and program acceptability are emailed to all individuals who register and attend a program as well as to walk-in participants who did not register but attended and provided their email address. SPs are offered to cancer survivors and caregivers across all cancer sites. Evaluations are reviewed after every program to measure the effectiveness in meeting the program goals and objectives and to identify trends in registration, attendance, and participant responses that inform necessary changes to the program.

### Analysis-

Descriptive statistics were utilized to summarize available data from the study period. The distribution of participant characteristics is presented using frequencies and percentages for categorical data and using means and standard deviations for continuous data. Zip code data is used to categorize counties of residence within the state of Ohio. Approval for use and publication of our institution’s internal quality data related to this study was granted by The Ohio State University Comprehensive Cancer Center (OSUCCC) – #2023COO46 and the James Quality and Patient Safety Committee.

## Results

The James and Stephanie Spielman Comprehensive Cancer Center treat 1,100 breast cancer patients each year. James*Care* for *Life* provides supportive and educational programs that are offered at no charge and available to individuals diagnosed with cancer and their caregivers/family members. Programs can be accessed from the time of diagnosis and throughout survivorship. James*Care* for *Life* programs facilitate the holistic treatment of breast cancer including the physical, emotional, spiritual, and practical aspects through education, healthy lifestyle programs and peer support groups. The James*Care* for *Life* program areas include Art and Music classes, Living Well with Advanced Breast Cancer, Survivorship Conference, Education, Exercise, Family, Teens, and Children, Mind, Body and Spirit, Nutrition, and Young Survivors. The programs are offered in person and online. Classes are facilitated by other cancer experts including physicians, nurses, dieticians, physical therapists, social workers and integrative medicine and wellness specialists. From 2019–2022, 2,198 breast cancer participants attended James*Care* for *Life* programs. The most attended programs during this period were nutrition and exercise. The annual percentage of participants attending nutrition programs ranged from 32.5% (2019) to 39.9% (2022). For exercise, the annual percentage of participants ranged from 13.7% (2019) to 22.6% (2022). Participation in the exercise program increased during the years of the COVID pandemic while participation in the nutrition program decreased. [Table 1]

Within the study period, a majority of James*Care* for *Life* attendees were White (74.0% - 82.3%). Black attendance decreased from 9.9% (2019) to 5.7% (2022). Asian/Pacific Islander attendees initially made up 4.3% of all attendees in 2019. During the COVID pandemic, their participation increased to 7.4% - 14.0% of all participants and then decreased to 8.7% in 2022. All other minority groups including American Indian, Hispanic/Latino, multi-racial, and other decreased during the COVID pandemic and through 2022. [Table 2]

From 2019–2022, the majority of James*Care* for *Life* attendees were within the 60–69 age group (45.8% - 60.1%). All age groups except 60–69 years-old and 70+ years age groups had an overall decrease in program participation in 2019 compared to 2022. Patients in the 18–39, 40–49, and 50–59 age ranges participated less during the COVID pandemic while 60–69 years-old and 70+ years-old groups had an increase in participation. Notably, participants age 70+ years-old nearly doubled from 9.4% (2019) to 18.1% (2022). [Table 3]

The James and Stephanie Spielman Comprehensive Breast Center offer additional clinical resources with referrals available to breast cancer patients. These clinical resources include Adolescent/Young Adult (AYA), Fertility Preservation, Palliative Care, Psychosocial Oncology (PSO) and Survivorship. These clinical resources are additional visits with a specialty physician or advanced practice provider (APP), or a mental health provider. A total of 5,297 patients were referred to these clinical resources in 4 years. The highest number of referrals were to the survivorship clinics (52%) followed by psychosocial oncology. Of all the clinical resources, the highest proportion of completed referrals were to the Adolescent/Young Adult (97%) and Fertility Preservation (74%) in 2019. The percentage of completed referrals out of total referrals remained the highest for Fertility Preservation in 2022 (80%). The percentage of referrals for Black patients to Palliative Care, Psychosocial Oncology, and Survivorship all increased from 2019 to 2022. Of note, Black patients were frequently referred to Palliative Care, with the proportion of referrals nearly doubling from 11% (2019) to 21% (2022). Referrals to all clinical resources increased for Latino/Hispanic patients from ≤1% in 2019 to 2–6% in 2022. [Table 4]

In 2019 most survivorship participants fell within the 65–74 age group (30%), however it was the 55–64 age group that saw the highest referral rate in 2022 (31%). Referrals to survivorship clinics in the 75+ age group saw a 64% decrease within the study period while referrals to palliative care saw a 175% increase. [Table 5]

**Figure 1:**
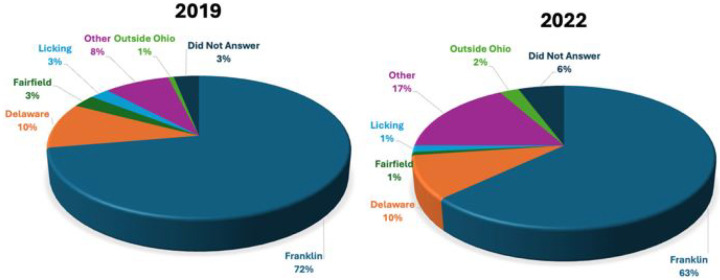
Attendance by County of Primary Residence: Total Number of Participant Attendees in Calendar Years 2019 and 2022

Annually, ~72% of the study population resided in Franklin County, followed by Delaware (up to 10%). [Figure 1]

## Discussion

These data show differences in provider referrals to clinical survivorship resources for different age groups and White compared to Black women. With patient attendance in non-clinical SPs, utilization is similar across race and age groups, with the majority choosing nutrition and exercise programs. Notable, however, is the decrease in Black participants over the study period declining from 9.9% (2019) to 5.7% (2022). This keeps with the observation that Black patients outside of minority serving institutions have lower participation in survivorship support services and programs [10,11,15]. Black women of lower socioeconomic status are also less likely to undergo guideline-concordant survivorship care [16]. Ko, et al recently investigated the unique needs of survivorship care of Black patients at multiple institutions and identified religion and spirituality as key resources for coping with breast cancer [17]. In this study, clinical referrals to palliative care increased for Black patients. Historically, Black patients present with more advanced disease and worse prognosis [1,18,19]. This may necessitate involvement of palliative care services that address pain management in addition to end of life planning. According to SEER data from 2017–2021, breast cancer patients aged 65–74 make up the majority of newly diagnosed female breast cancer, with a median age at diagnosis of 63 years-old [20]. Elderly breast cancer patients commonly present with concurrent increased frailty, comorbid conditions, decreased functional status, and fewer social and economic resources.

The unique needs of this vulnerable majority of breast cancer survivors have not been well studied [21,22]. The elderly patients in our study are getting fewer clinical referrals to survivorship, however, they are increasing their participation in James*Care* for *Life* programs. It appears they are self-selected for more support services; however, providers are not referring them for a formal survivorship visit as often. In a study by Krok-Schoen et al. analyzing the perspectives of survivorship care plans (SCP) among older breast cancer survivors, while all patients received SCPs, less than a quarter of them were aware of these plans suggesting a communication barrier between patients and their providers. Suggested areas of improvement included clearer communication, more long-term resources, and the use of health coaches to facilitate patients’ adherence to their SCPs [23].

To reduce barriers and facilitate access to survivorship care and services, navigation is increasingly utilized. Although historically associated with screening, navigation can be used across the breast cancer care continuum [24]. Insurvivorship, a navigator can educate women on how to improve their overall wellness, thereby directly impacting the health of a growing population of cancer survivors [25–28]. Studies show that navigation has increased patient reported quality of life [29, 30]. Dixit et al. show trends towards improvement in the change in emotional well-being score, functional well-being score and overall QOL in the intervention arm, but it was not statistically significant. The change in physical well-being scores was similar. There was no difference in the mean self-efficacy scores for both arms [30]. In a recent randomized trial of Hispanic breast cancer survivors, Ramirez et al. showed that navigation resulted in significantly improved quality of life measures in a 6‐month period [31]. Unfortunately, most of these studies did not include Black or elderly women.

Establishing survivorship programming for the breast cancer population requires consideration of disparities in patient ages, race and ethnicity, and education among other variables. Monitoring and evaluating these data offerings allow programming tailored to the population. At our institutions patients are majority White and reside within our local catchment (Franklin County), despite our patient catchment extending across the entire state of Ohio and beyond to neighboring states of West Virginia, Indiana, and Kentucky. Therefore, we are not attracting the wider segment of our breast cancer survivors. Even with most of our SPs transitioned to virtual visits, attendance has not broadened beyond Franklin County. There is considerable dynamism with the county population necessitating frequent evaluation of SPs, new immigrant communities and non-native English speakers have changed the local demographic over the years. This will require expansion of programs to Spanish language focus for example. Our current study shows only ~2% of our participants self-reported Hispanic ethnicity which will increase as the local population evolves.

### Limitations

Given the retrospective nature of this study anticipated biases are present. Also, participant self-reported data is at risk for recall bias. Furthermore, the database used is focused on quality evaluation and is therefore limited in terms of the data variables captured. While this manuscript is focused on “patients” and being referred to supportive care resources, JCFL programs aren’t just available for James patients but are also open to community members who might not be James patients and might not have been referred to programs by their healthcare team. This is a limited number but is not differentiated by the current database. Similarly, planned deeper analysis to explore factors associated with participation was limited due to the lack of collected anonymous and individual data. Qualitative data is lacking that would allow for a better understanding of why certain programs were selected and what future individual needs are.

## Conclusion

These descriptive data show the population our current survivorship service line serves and associated participant preferences. Black women underutilize these supportive services, while women over 70 years-old have increased their participation. Further investigation is planned to explore and understand factors associated with SP participation. A necessary first step is to optimize the institutional database to improve post participation data capture. Future program development will address inclusivity of minority and elderly women, as well as ensure equitable access to survivorship resources through navigation.

## Figures and Tables

**Table 1.** James*Care* for *Life* Attendance by Program Area: Total Number of Participant Attendees in Calendar Years 2019–2022

Program Area	Total Breast Cancer Participants – CY2019	Total Breast Cancer Participants – CY2020	Total Breast Cancer Participants – CY2021	Total Breast Cancer Participants - CY2022
Art	24(5.8%)	24(4.6%)	16(2.7%)	14(2.6%)
Ask the Expert – Living Well with Advanced Breast Cancer	13(3.1%)	29(5.5%)	NA – Program on Hold	18(3.3%)
Breast Cancer Survivorship Conference	78(18.8%)	61(11.6%)	41(6.8%)	44(8.1%)
Education	41(9.9%)	41(8.0%)	59(9.8%)	55(10.2%)
Exercise	57(13.7%)	168(32.1%)	227(37.7%)	122(22.6%)
Family, Teens, Children	13(3.1%)	4(0.8%)	5(0.8%)	1(0.2%)
Mind, Body, Spirit	41(9.9%)	65(12.4%)	103(17.1%)	66(12.2%)
Music	7(1.7%)	4(0.8%)	4(0.7%)	5(0.9%)
Nutrition	135(32.5%)	121(23.1%)	142(23.6%)	216(39.9%)
Young Survivors	6(1.4%)	6(1.1%)	5(0.8%)	0(0.0%)

**Table 2.** James*Care* for *Life* Attendance by Participant Race/Ethnicity: Total Number of Participant Attendees in Calendar Years 2019–2022

Program Participant Race/Ethnicity	Total Breast Cancer Participants – CY2019	Total Breast Cancer Participants – CY2020	Total Breast Cancer Participants - CY2021	Total Breast Cancer Participants – CY 2022
African American/Black	41(9.9%)	43(8.2%)	40(6.6%)	31(5.7%)
American Indian	4(1.0%)	0(0.0%)	1(0.2%)	1(0.2%)
Asian/Pacific Islander	18(4.3%)	39(7.4%)	84(14.0%)	47(8.7%)
Caucasian/White	307(74.0%)	409(78.1%)	448(74.4%)	445(82.3%)
Hispanic/Latino	7(1.7%)	5(1.0%)	0(0.0%)	4(0.7%)
Multi-racial	14(3.4%)	10(1.9%)	6(1.0%)	6(1.1%)
Other	19(4.6%)	15(2.9%)	23(3.8%)	2(0.4%)
Did not answer	5(12%)	3(0.6%)	0(0.0%)	5(0.9%)

**Table 3.** James*Care* for *Life* Attendance by Participant Age: Total Number of Participant Attendees in Calendar Years 2019–2022

Program Participant Age	Total Breast Cancer Participants – CY2019	Total Breast Cancer Participants – CY2020	Total Breast Cancer Participants - CY2021	Total Breast Cancer Participants – CY 2022
18–39 years old	25(6.0%)	17(3.2%)	15(2.5%)	20(3.7%)
40–49 years old	53(12.8%)	47(9.0%)	65(10.8%)	35(6.5%)
50–59 years old	79(19.0%)	99(18.9%)	88(14.6%)	87(16.1%)
60–69 years old	190(45.8%)	315(60.1%)	324(53.8%)	262(48.4%)
70+ years old	39(9.4%)	32(6.1%)	82(13.6%)	98(18.1%)
Did not answer	29(7.0%)	14(2.7%)	28(4.7%)	39(7.2%)

**Table 4.** Clinical Resource Referrals by Race: Total Number of Participants Referred to Clinical Resources in Calendar Years 2019 and 2022

Clinical Resource	2019									
	*Total Referrals*	*Completed Referrals*	*%*	*Race*				*Ethnicity*		
				*Black*	*White*	*Asian*	*Other*	*Not Hispanic or Latino*	*Latino/Hispanic Other*	*Unknown*
AYA	38	37	**97%**							
Fertility Preservation	38	28	**74%**		99%		1%	99%	1%	
Palliative Care	112	64	**57%**	11%	89%			99%		1%
Psychosocial Oncology	379	190	**50%**	11%	84%	1%	4%	99%	1%	
Survivorship (Survivorship, Sexual Health)	797	546	**69%**	9%	85%	2%	4%	98%	1%	1%
AYA	116	60	**52%**	13%	83%	2%	2%	96%	2%	2%
Fertility Preservation	20	16	**80%**	13%	68%		19%	94%	6%	
Palliative Care	120	63	**53%**	21%	74%		5%	97%	3%	
Psychosocial Oncology	401	212	**53%**	17%	76%	3%	4%	98%	2%	
Survivorship (Survivorship, Sexual Health)	584	372	**64%**	14%	81%	1%	4%	97%	2%	1%

**Table 5.** Clinical Resource Referrals by Age: Total Number of Participant Referred to Clinical Resources in Calendar Years 2019 and 2022

CY 2019							
	25–34	35–44	45–54	55–64	65–74	75+	TOTALS
AYA	-	-	-	-	-	-	-
Fertility	2(11.1%)	10(55.6%)	4(22.2%)	2(11.1%)	0 (0.0%)	0(0.0%)	18(2.2%)
Palliative	1(2.1%)	6(12.8%)	10(21.2%)	14(29.8%)	12(25.5%)	4(8.5%)	47(5.8%)
PSO	9(4.7%)	24(12.6%)	59(31.1%)	53(27.9%)	34(17.9%)	11(5.8%)	190(23.6%)
Survivorship	5(0.9%)	32(5.8%)	130(23.6%)	149(27.1%)	164(29.8%)	70(12.7%)	550(68.3%)
CY 2022							
	23–34	35–44	45–54	55–64	65–74	75+	TOTALS
AYA	21(31.8%)	43(65.5%)	2(3.0%)	0(0.0%)	0(0.0%)	0(0.0%)	66(8.6%)
Fertility	10(47.6%)	11(52.4%)	0(0.0%)	0(0.0%)	0(0.0%)	0(0.0%)	21(2.7%)
Palliative	2(2.8%)	13(18.1%)	11(15.3%)	17(23.6%)	18(25.0%)	11(15.3%)	72(32.4%)
PSO	11(4.4%)	44(17.7%)	75(30.1%)	60(24.1%)	46(18.5%)	13(5.2%)	249(32.4%)
Survivorship	7(1.9%)	42(11.6%)	75(20.8%)	111(30.7%)	101(28.0%)	25(6.9%)	361(46.9%)
